# *Listeria monocytogenes* in Ready-to-Eat Foods: Risk Perspectives Across Different Regulatory Systems

**DOI:** 10.3390/foods15030470

**Published:** 2026-01-29

**Authors:** Giovanni D’Ambrosio, Maria Schirone, Antonello Paparella

**Affiliations:** Department of Bioscience and Technology for Food, Agriculture and Environment, University of Teramo, Via Balzarini 1, 64100 Teramo, Italy; gdambrosio@unite.it

**Keywords:** *Listeria monocytogenes*, regulations, microbiological criteria, risk, food safety management

## Abstract

*Listeria monocytogenes* poses a significant challenge in ready-to-eat (RTE) foods due to its persistence in processing environments and severe impact on vulnerable populations. Regulatory approaches differ internationally, reflecting distinct conceptual frameworks and tolerance thresholds. These differences arise from the adoption of zero-tolerance or risk-based regulatory models, which define qualitative or quantitative microbiological limits (absence in 25 g or up to 100 cfu/g) based on a product’s growth potential, and vary in the extent of environmental monitoring, sampling plans, and verification intensity across jurisdictions. In 2024, the European Union updated its regulatory framework governing the microbiological criteria for *L. monocytogenes*. Previous requirements were strengthened, responsibility was extended across the supply chain, and a strategic role was assigned to challenge testing carried out by manufacturers. This review examines how the European Union and the United States apply risk assessment principles, challenge testing, predictive modelling, and environmental monitoring to control *L. monocytogenes* in RTE foods. By integrating epidemiological trends, regulatory criteria, and experimental evidence, key differences in safety objectives, validation procedures, and risk management strategies are highlighted. This review also identifies gaps and opportunities for harmonisation, providing guidance for improved evidence-based decision-making and regulatory compliance.

## 1. Introduction

*Listeria monocytogenes* is one of the most critical foodborne pathogens associated with ready-to-eat (RTE) foods, which represent the primary vehicle for human listeriosis due to the absence of a final listericidal treatment and the microorganism’s ability to survive and proliferate under conditions that inhibit many other pathogens. Key physiological traits—including growth at refrigeration temperatures, tolerance to relatively low pH and water activity (a_w_), and strong biofilm-forming capacity on food-contact surfaces (FCSs)—favour long-term persistence in food-processing settings and dissemination along the food chain [1]. High-risk RTE foods include pre-prepared salads, deli meats, cold-cooked poultry, soft cheeses, and cold-smoked or marinated fish. These food products combine permissive intrinsic properties (a_w_ > 0.92, pH ≥ 5.0) with post-processing handling, slicing, and repackaging, which can promote cross-contamination or pathogen growth if hygiene practices are inadequate [2]. The risk is amplified by the fact that RTE foods are generally consumed directly, without further processing by the consumer. Moreover, vacuum or modified atmosphere packaging allows *L*. *monocytogenes* growth during refrigerated storage [3]. Contamination levels in RTE foods typically range from very low (<1 cfu/g) up to levels approaching the regulatory limit of 100 cfu/g at the end of their shelf life [4].

Although human listeriosis is relatively rare, its symptoms can be severe, making even minimal contamination in RTE foods a significant public health concern [5]. Nearly all patients are hospitalised, with a case-fatality rate of approximately 20% in the general population; about 25% of pregnancy-associated cases result in foetal loss or neonatal death [6]. The latter statistic underscores the heightened vulnerability of certain groups, including the YOPI category (Young, Old, Pregnant, Immunocompromised) [7]. Listeriosis occurs in two main forms: non-invasive, with gastroenteritis and mild fever in healthy adults, and invasive, which occurs mainly in the YOPI population. Invasive listeriosis affects the nervous system, with sepsis, meningitis, meningoencephalitis, miscarriage, and perinatal infections [8]. Incubation can last up to three months, thus complicating diagnosis and treatment.

Diagnosis of listeriosis relies on the integration of information from multiple sources, including clinical cases, food monitoring data, animal isolates, and environmental sampling. For this reason, listeriosis represents one of the earliest and most relevant applications of the One Health framework, playing a central role in prevention strategies both in clinical and food-related settings. Conventional diagnostic tools, including culture-based methods, polymerase chain reaction (PCR), and matrix-assisted laser desorption ionisation time-of-flight mass spectrometry (MALDI-TOF MS), are routinely employed for the detection and preliminary identification of *L*. *monocytogenes*. However, these approaches are often constrained by lengthy turnaround times, limited sensitivity for low-level or stressed cells, and insufficient discriminatory power to reliably distinguish closely related strains or establish robust epidemiological links. Consequently, their utility in outbreak investigations, source attribution, and persistence tracking is limited [9]. In contrast, Whole Genome Sequencing (WGS) has emerged as the gold standard for *L*. *monocytogenes* surveillance and control. By providing high-resolution strain typing, WGS enables precise integration of clinical, food, and environmental data, thereby enhancing epidemiological investigations and contamination source identification in food-processing and retail contexts. In addition, WGS allows in-depth characterisation of genetic determinants associated with virulence, stress adaptation, and environmental persistence, strengthening risk assessment and supporting more targeted, effective control strategies [10].

Recent European data show an upward trend in invasive listeriosis. In 2024, 3041 confirmed cases were reported across 26 EU Member States (0.69 per 100,000 population), with 301 fatalities, predominantly among older adults [11]. To illustrate the recent trend of invasive listeriosis in the EU, Table 1 reports the annual number of human cases and notification rates from 2020 to 2024.

In the United States (US), listeriosis affects approximately 1250 people annually, resulting in over 1000 hospitalisations and around 170 deaths [16]. Between 2018 and 2023, 26 foodborne outbreaks were reported, causing 222 illnesses, 200 hospitalisations, and 25 deaths. Improvements in outbreak detection, molecular subtyping, and the implementation of preventive control measures likely contributed to the lower number of cases per outbreak during this period [17].

Among many factors, the growing number of cases is favoured by the increasing consumption of RTE products [3,18]. This apparent discrepancy between generally low contamination levels in RTE foods and rising human cases underscores the need for robust tools to assess whether foods can support pathogen growth during storage. Microbial challenge studies provide a direct experimental evaluation of growth, survival, or inactivation, taking into account the complex interactions between microbial physiology, the food matrix, and storage conditions [19,20]. Such studies differ from conventional shelf life testing because they deliberately investigate microbial behaviour under controlled contamination scenarios [21].

Challenge testing has gained further importance due to recent regulatory developments. In the European Union (EU), Regulation (EU) No. 2024/2895 [22], amending Regulation (EC) No. 2073/2005 [23], requires that RTE foods capable of supporting *L. monocytogenes* growth ensure absence in 25 g throughout their shelf life, unless validated evidence demonstrates that levels will remain below 100 cfu/g until consumption. In this respect, international approaches differ. For example, in the US, the FDA (Food and Drug Administration) and USDA-FSIS (United States Department of Agriculture—Food Safety and Inspection Service) adopt risk management frameworks that diverge from the European model, as they define distinct tolerance limits and validation requirements. Awareness of these differences is essential for interpreting challenge study data and contextualising their application across regulatory systems.

Previous reviews have only analysed a few specific regulatory aspects, such as public health and business risks [24], the European guidelines for challenge testing [25,26], technical issues in challenge tests [27], the evolution of RTE food regulations [28], and changes in the European regulations on microbiological criteria [29]. To the best of our knowledge, no comparative study has been carried out regarding the different legislative perspectives and methodological approaches employed towards the control of *L*. *monocytogenes* in RTE foods. This research gap limits the opportunity to improve evidence-based decision-making, regulatory compliance, and ultimately, risk management. Therefore, this review aims to compare EU and US regulations on *L. monocytogenes* in RTE foods to discuss the regulatory interpretation of challenge testing, considering the factors that affect pathogen behaviour, the methodological differences, and the role of challenge tests in risk management.

## 2. Methods

This review was prepared through an ordered succession of activities. Firstly, the main issues of the topic were identified, including regulations on RTE foods and *L. monocytogenes*, methods for challenge testing, and differences between the EU and the US. Secondly, publications were extracted by different search engines (Scopus, PubMed, Google Scholar, and ResearchGate) from 1 November 2025 to 15 December 2025, using the following keywords: “regulation”, “*Listeria monocytogenes*”, “challenge test”, “criteria”, “limit”, “risk”, “public health”, “RTE”, “food”, and “review”. Combinations and permutations of these keywords were made to gain a comprehensive analysis of the scientific literature. Then, publications were analysed and skimmed based on their relevance and coherence with the topic and quality of data. In this step, similarities and differences among the sources were highlighted. Full-text articles needed to include analytical observations about the two different regulatory approaches, respective regulations, and information regarding the prevalence of *L*. *monocytogenes* in RTE foods, along with challenge testing applied to them. Moreover, technical guidance documents regarding challenge testing and environmental monitoring for *L*. *monocytogenes*, by both the EU and the US, were included for writing the review. The grey literature in the form of PhD theses and white papers was excluded from this review for feasibility reasons, due to the large amount of published data and usually the absence of a formal peer review. Finally, the results were organised into sections, starting with the organisation of the challenge test and then considering its role in EU and US regulations, as well as its impact on risk management.

## 3. Conceptual Basis of Microbiological Challenge Testing

Microbiological challenge testing provides a controlled experimental framework to assess the behaviour of *L*. *monocytogenes* in real food systems under defined storage conditions. Unlike approaches based solely on physical and chemical parameters, challenge tests integrate product composition, microbial traits, and environmental factors, allowing direct evaluation of growth potential, survival, or inactivation throughout the intended shelf life [30].

### 3.1. Product-Related Constraints

The fate of *L*. *monocytogenes* in RTE foods is influenced by the combination of matrix characteristics and storage conditions. Factors such as a_w_, pH, antimicrobial constituents, redox potential, and the presence of background microbiota collectively define the ecological niche encountered by the pathogen. While growth thresholds based on a_w_ and pH are well established, they may be insufficient to predict microbial behaviour in real foods, where spatial heterogeneity, microenvironments, and temporal changes can modulate survival and growth. Nutrient availability, redox conditions, and interactions with resident microbiota further influence pathogen dynamics, often limiting proliferation despite seemingly permissive conditions [31]. Therefore, experimental verification within the specific food matrix remains essential.

### 3.2. Biological Relevance of the Inoculum

The outcome of a challenge study is strongly influenced by the characteristics of the inoculum. Initial contamination levels should reflect realistic scenarios while remaining analytically robust. In fact, overly low inoculum levels may mask growth, whereas excessively high levels can overwhelm the inhibitory capacity of the matrix. The physiological state of the microorganism, including culture history and stress adaptation, also affects lag phase duration and growth kinetics. Standardised preparation and transparent reporting of strain conditioning are necessary to ensure reproducibility and biological relevance [32].

### 3.3. Strain Selection and Inoculation Strategy

Selecting appropriate strains is a crucial design consideration. Isolates from relevant food types or processing environments are preferred, as they better reflect adaptation to product-specific stresses, especially in mild stress conditions [33]. When reference strains are used, their representativeness should be justified. Inoculation should achieve a homogenous distribution while minimising impacts on product characteristics. Using strain cocktails can enhance representativeness, but potential interactions among strains must be considered when interpreting results [34].

### 3.4. Storage Conditions and Study Duration

Challenge tests should cover the full intended shelf life and replicate realistic storage conditions, including distribution, retail, and domestic handling, as well as packaging atmosphere. Therefore, the simulation of storage conditions may involve the use of equipment that is not commonly available in a microbiology lab, such as packaging machines. When operations such as cutting, dosing, and packaging cannot be performed in the lab, inoculation should be carried out through the original packaging, using a procedure that ensures sterility, maintenance of the packaging conditions, and inoculum distribution. Microbial kinetics primarily depend on temperature, and even slight variations can significantly change the results. Including limited, realistic variations may help capture foreseeable variability, provided these are defined in the study design [35].

### 3.5. Interpretation of Outcomes

**Figure 1:**
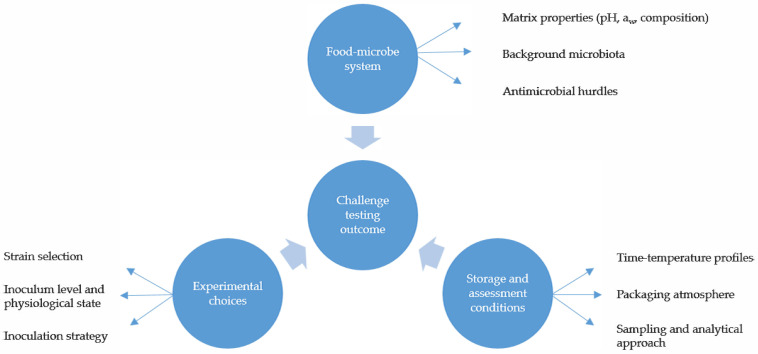
Main elements influencing the outcomes of microbiological challenge testing for *L*. *monocytogenes* in RTE foods.

Sampling schemes must be sufficient to evaluate temporal trends, beginning with the verification of initial contamination levels. Replication and appropriate controls are essential for distinguishing true inhibitory effects from methodological artefacts. Results should be interpreted in conjunction with measurements of relevant product parameters and predefined acceptability criteria. Rather than serving as isolated observations, challenge test outcomes provide evidence to support product safety, shelf life validation, and regulatory compliance [36]. The main elements influencing the outcome of microbiological challenge testing and their interrelationship are summarised in Figure 1.

## 4. Regulatory Frameworks for *L. monocytogenes* in RTE Foods

The control of *L*. *monocytogenes* is governed by distinct regulatory frameworks in the EU and the US, with significant differences in risk philosophy, legal definitions, and compliance requirements. Effective regulation is essential to mitigate public health risks, particularly given the pathogen’s ability to persist in processing environments and proliferate under refrigeration. This section provides an overview of international approaches, followed by a detailed discussion of EU and US legislation.

### 4.1. European Regulations

Within the EU, *L*. *monocytogenes* is regulated by Regulation (EC) No. 2073/2005 [23] and its subsequent amendments, including Regulation (EU) No. 2024/2895 [22]. These regulations establish microbiological criteria applicable throughout the product’s shelf life and define RTE foods as products intended for direct human consumption without further cooking or processing capable of eliminating or reducing microorganisms of concern.

Compliance is based on the food category and target consumer group, with limits ranging from absence in 25 g to a maximum of 100 cfu/g at the end of shelf life. The quantitative limit of 100 cfu/g applies exclusively to RTE foods not supporting the growth of *L*. *monocytogenes* and not intended for infants or special medical purposes. According to Regulation (EC) No. 2073/2005 [23], RTE foods are considered non-growth-supporting when they meet at least one of the following conditions: (i) pH ≤ 4.4; (ii) a_w_ ≤ 0.92; (iii) a combination of pH ≤ 5.0 and a_w_ ≤ 0.94; or (iv) a shelf life shorter than five days. Other products may also be classified as non-growth-supporting if substantiated by scientific evidence, such as challenge testing or predictive modelling. For RTE foods capable of supporting growth, Regulation (EC) No. 2073/2005 [23] requires the absence of *L*. *monocytogenes* in 25 g at the end of shelf life. The recent amendment introduced by Regulation (EU) No. 2024/2895 [22], effective July 2026, clarifies and reinforces these requirements, allowing a quantitative limit of 100 cfu/g only if it can be scientifically demonstrated that the pathogen will remain below this threshold throughout the product’s shelf life. The updated framework places greater emphasis on strengthened self-monitoring systems, environmental and raw material surveillance, definition of intermediate control limits, continuous staff training, reinforced cold-chain management, and the use of predictive modelling and challenge testing tools to demonstrate compliance [37].

The acceptance of a quantitative limit for non-growth-supporting RTE foods reflects the practical difficulty of ensuring the complete absence of the pathogen in products subjected to mild processing or post-lethality handling. This regulatory choice is supported by risk assessment conclusions indicating that concentrations below 100 cfu/g at consumption pose a low risk for non-vulnerable consumers [38].

European legislation does not prescribe detailed or harmonised requirements for environmental monitoring of *L*. *monocytogenes* or *Listeria* spp. The only explicit legal obligation is contained in Article 5(2) of Regulation (EC) No. 2073/2005 [23], which requires food business operators (FBOs) producing RTE foods at risk of listeriosis to carry out environmental sampling of processing areas and equipment. Additional guidance has been provided through non-binding documents, including the Codex Alimentarius guidelines on the control of *L*. *monocytogenes* in RTE foods [39], which recommend implementation of environmental monitoring, particularly during plant modifications or renovations. Further operational guidance was issued by the EU Reference Laboratory for *L*. *monocytogenes* (EURL Lm) in collaboration with ANSES [40]. Earlier versions of this document explicitly recognised *Listeria* spp. as an indicator of conditions supporting *L*. *monocytogenes* growth when detected on FCSs [41]. However, this point was removed in subsequent revisions, reflecting the absence of an aligned European position on indicator-based environmental monitoring. Thus, the implementation of environmental monitoring programmes remains largely operator-driven, despite evidence supporting the value of systematic testing for *Listeria* spp. as an early warning tool [42,43].

Mandatory environmental testing requirements primarily arise in the context of exports to the US. In fact, European establishments authorised to export RTE foods, particularly meat products, shall comply with supplementary control measures under US equivalence determinations, including enhanced sanitation procedures and environmental sampling aligned with USDA-FSIS expectations [44,45].

According to Regulation (EC) No. 2073/2005 [23], sampling frequencies for finished products are not fixed, and FBOs are encouraged to define sampling plans based on HACCP principles and Good Hygiene Practices (GHPs). Specific minimum sampling frequencies apply only to certain raw materials, with provisions allowing reduced sampling when sustained compliance is demonstrated. To support compliance with these requirements, structured food safety management tools (e.g., FMEA, HAZOP, and Ishikawa diagrams) and modelling of time–temperature effects during storage and distribution have been proposed [46,47,48], although further research is needed to assess their practical implementation.

### 4.2. United States Regulations

In the US, food legislation is administered by two federal agencies: the FDA regulates most foods, including seafood, dairy, and non-FSIS-regulated meats [49], while the USDA-FSIS oversees meat, poultry, and egg products. The legal framework is founded on the Federal Food, Drug, and Cosmetic (FD&C) and the Federal Meat Inspection (FMI) Act for FSIS.

Prior to the early 1980s, listeriosis was primarily regarded as an animal-associated disease, mainly affecting livestock such as sheep and cattle. This perception changed following a series of severe foodborne outbreaks in North America, beginning with the 1981 coleslaw outbreak [50] and culminating in the Jalisco cheese outbreak [51]. These events, characterised by high case-fatality rates, particularly among vulnerable populations [52], established *L*. *monocytogenes* as a ubiquitous foodborne pathogen and exerted substantial pressure on regulatory authorities. In response, the FDA adopted a “zero-tolerance” policy for this pathogen in RTE foods [53], whereby any detectable presence is considered a public health hazard. This policy has since become a cornerstone of the US regulatory approach and continues to shape hazard control strategies for RTE foods. Under FDA oversight, a food is considered adulterated if *L*. *monocytogenes* is present [54,55]. This reinforces the principle that contamination should be preventable through the application of Good Manufacturing Practices (GMPs) and sanitation [52]. Despite the introduction of risk-informed preventive controls under the Food Safety Modernisation Act (FSMA), a zero-tolerance policy remains in place. This confirms ongoing concerns related to strain variability, host susceptibility, and the severity of listeriosis.

The USDA-FSIS applies a similar legal concept for meat and poultry products, with zero-tolerance through mandatory HACCP systems, sanitation regulations, and the “*Listeria* Rule” (9 CFR §430), which specifically addresses the risk of post-lethality contamination [56]. Regulatory emphasis is placed on environmental control rather than routine end-product testing. The detection of *Listeria* spp. on FCSs is interpreted as evidence of conditions that may permit contamination, which trigger corrective actions, whereas finished product testing is required only under specific “hold-and-test” scenarios. Consistent with this preventive orientation, US industry practice has widely adopted proactive environmental investigation strategies such as the “Seek and Destroy” (S&D) approach. As originally described by Butts [57], S&D aims to identify and eliminate persistent growth niches and harbourage sites in food-processing environments. This approach distinguishes between transient contamination points, true growth niches capable of supporting microbial proliferation after sanitation, and persistent harbourage sites. In detail, S&D relies on targeted equipment disassembly, focused environmental sampling, and multidisciplinary team involvement. By facilitating root-cause analysis and evaluation of sanitary design deficiencies, S&D aligns closely with the zero-tolerance philosophy and supports the control of post-lethality contamination [58].

A further distinguishing feature of the US regulatory system concerns the definition of RTE food. FSIS primarily relies on product labelling and the absence of cooking or safe-handling instructions to classify RTE status, while the FDA applies a broader reasonably foreseeable consumption criterion, which accounts for consumer behaviour irrespective of manufacturer intent. These differences underscore the preventive, hazard-focused orientation of US regulation and further distinguish it from the risk-based European approach. To provide a broader perspective, Table 2 presents a comparison of risk management approaches and regulatory criteria for *L. monocytogenes* in several countries, including Australia, China, and Japan.

## 5. Role of Challenge Testing in Risk Management and Hazard Control

In hazard-based regulations, as in the US, the mere detectable presence of a pathogenic agent can trigger regulatory action, exemplified by the zero-tolerance policy for *L*. *monocytogenes* in RTE foods. While this approach offers high consumer protection, it may be overly conservative for foods that do not support pathogen growth or where exposure is low [67]. In contrast, risk-based approaches integrate data on pathogen prevalence, concentration, exposure, and host susceptibility to estimate risk and guide decision-making, as in the EU regulations on *L. monocytogenes* [68].

Quantitative Risk Assessment (QRA), which encompasses hazard identification, hazard characterisation, exposure assessment, and risk characterisation, underpins European food safety policy and supports the Food Safety Objective (FSO) of 100 cfu/g for *L*. *monocytogenes* in certain RTE foods [67]. Limitations of this approach include data requirements, technical complexity, and uncertainties in exposure assessment. These conceptual differences, summarised in Table 3, influence goals and the regulatory application of challenge testing.

In the EU, challenge studies primarily validate shelf life and categorise products as growth-supporting or non-growth-supporting [69]. In the US, challenge testing serves to evaluate growth potential, validate growth inhibitors, and document the effectiveness of control measures under zero-tolerance expectations [70]. However, even if a growth criterion value is given for the EURL Lm, the FDA, and the USDA-FSIS challenge test, in order to consider an RTE product as a growth-favourable substrate or not, these values are not directly comparable. In fact, while the EURL Lm growth potential (δ) refers to a net growth, as it is the difference between the highest load throughout the shelf life and the initial load, the US values refer to the absolute growth of *L*. *monocytogenes* throughout the shelf life. Consequently, study design, endpoints, and interpretation criteria differ between jurisdictions. Differences between challenge testing methodologies are given in Table 4.

**Figure 2:**
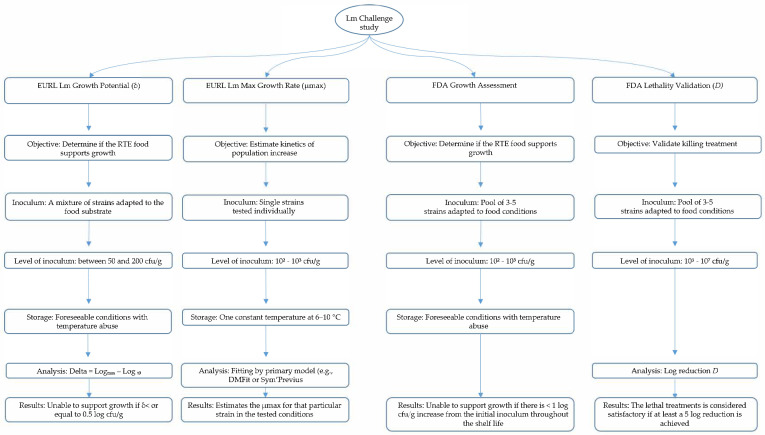
Main steps involved in the EURL Lm growth potential and maximum growth rate, and the FDA growth assessment and lethality validation challenge studies.

A summarised comparative flowchart regarding the main steps of the EU and US challenge test is shown in Figure 2.

Although neither the EU nor the US prescribes fixed frequencies for challenge testing, studies are required for initial product validation, formulation changes, or implementation of new control measures and may be repeated following monitoring failures or periodic verification [59,60]. Challenge testing for *L. monocytogenes* can be extremely important in specific conditions, for example, to assess the safety of RTE raw meat products, especially when preservatives are removed and emerging technologies or ingredients are used [73]. Detailed technical guidance, including standardised laboratory methods and WGS for source attribution, is provided by US agencies [74,75]. A comparison between laboratory methods used in the EU [76,77] and the US is given in Table 5.

In a survey carried out in Italy on 161 establishments, of which 81 were authorised for the export of pork meat products to the US and 83 were only authorised for EU trade, no significant differences in consumer exposure between the two groups of establishments were found [44]. Nevertheless, the relative influence of regulatory systems, control measures, and biological variability remains poorly characterised, highlighting the need for further investigation. In the meantime, the differences between EU and US regulations on *L. monocytogenes* imply additional costs and business risks for EU companies exporting to the US, due to the zero-tolerance imposed by US regulations.

## 6. Technical Competence of Analytical Laboratories

Both EU and US guidelines for challenge testing related to *L. monocytogenes* in foods require analytical laboratories to have specific skills and organisation due to the complexity of the procedure. In fact, the challenge test cannot be considered a single reference method but rather a combination of procedures and reference methods, involving samples and inoculum preparation, microbiological analyses, physical and chemical measurements, and statistical analysis. In the EU, to ensure that laboratories have the necessary expertise and technical competence, the EURL Lm has issued a guidance document [78]. This guide aims to evaluate whether the laboratory can fulfil an important requirement for the *L. monocytogenes* criterion, which is the ability to demonstrate, to the satisfaction of the competent authority, that the product will not exceed the limit of 100 cfu/g throughout the shelf life.

Unlike the EU regulations on HACCP, where laboratory accreditation according to ISO/IEC 17025 [79] is required, challenge testing is not explicitly subjected to the same requirement. In fact, challenge tests cannot be assimilated to HACCP activities, as they can be carried out for different purposes, e.g., research, export, or new product development. In the EU, challenge testing for *L. monocytogenes* can be accredited on a voluntary basis. In Italy, to date [80], 14 laboratories have been accredited for challenge testing with the EURL Lm procedure according to ISO/IEC 17025.

On the other side, in the US, the laboratories that wish to obtain accreditation for challenge testing on *L. monocytogenes* need to adhere to a voluntary programme for laboratories that perform food analyses for FDA-directed purposes. In the so-called Laboratory Accreditation for Analysis of Foods Programme (LAAF Programme), the FDA recognises accreditation bodies that accredit laboratories for challenge testing according to ISO/IEC 17025. The recent advancements in the development of detection methods are accelerating the shift from the laborious and time-consuming conventional culture methods to rapid and sensitive technologies, which are cost-effective and specific, reducing the number of false positives [81]. Newly available detection methods include

- portable biosensing devices (such as smartphone-based biosensors), capable of detecting foodborne pathogens through colorimetric, fluorescence, or amperometric changes [82];
- CRISPR-Cas-based diagnostic tools, which enable high throughput, high sensitivity, and specificity even in composite foods [83];
- spectrometric approaches (such as MALDI-TOF MS), with their fast and high rates of detection, which could be of great use as an antimicrobial resistance (AMR) screening tool [84].

Table 6 briefly summarises and compares conventional methods with the most recent ones.

## 7. Conclusions and Future Perspectives

The effective management of *L*. *monocytogenes* in RTE foods requires translating scientific evidence into regulatory decisions that are both protective and proportionate to risk. EU risk-based and US hazard-based frameworks differ in philosophy, yet both aim to prevent severe listeriosis and minimise consumer exposure. These differences shape how experimental evidence—including challenge testing and environmental monitoring—is interpreted within regulatory decision-making. Challenge testing serves as a critical interface between science and regulation, supporting not only compliance but also informed decisions on shelf life, process control, and product formulation. However, differences in regulatory expectations between jurisdictions limit the direct comparability of results and can increase uncertainty for FBOs operating across multiple markets.

Future improvements will depend on integrating challenge testing with predictive tools to enhance consistency, transparency, and reliability in risk management. While tailorable models, such as gamma-based concept models, are already recommended for use in challenge testing for food products, as specified in ISO 20976-1:2019 [85], adoption remains limited due to the lack of consistent mathematical, biological, and statistical expertise among researchers, FBOs, and risk managers. Therefore, in order to enable the systematic use of predictive modelling tools, ease of use, results interpretability, and user-friendly interfaces must be ensured. Regarding this matter, the integration of artificial intelligence (AI) and machine learning (ML) technologies offers the potential to analyse large datasets, identify correlations, and improve microbial risk assessment [86]. AI- and ML-based tools could also overcome limitations encountered in conventional challenge testing, such as single-cell variability, genetic variations, and experimental parameter definitions [86,87]. Greater alignment between regulatory systems—based on shared principles for risk assessment and interpretation of experimental evidence, rather than uniform legal limits—represents a realistic pathway to strengthen food safety, support innovation, and facilitate international trade in the RTE sector.

Future research could test the following hypotheses: (i) overly restrictive or highly targeted SSOPs do not provide significantly greater control of *L*. *monocytogenes* compared to standard sanitation procedures; (ii) routine testing for *Listeria* spp. may not always improve risk management outcomes; and (iii) the “zero-tolerance” approach does not necessarily achieve higher consumer protection than a risk-based limit of 100 CFU/g. Further observational studies comparing the effectiveness of different sanitation procedures and environmental monitoring programs are needed to evaluate whether stricter food safety criteria can meaningfully reduce listeriosis incidence and overall case numbers. Continuous evaluation of emerging technologies, novel food ingredients, and consumer behaviours will also be essential to adapt regulatory strategies and maintain protection against listeriosis in evolving food landscapes.

## Figures and Tables

**Table 1:** Listeriosis in EU Member States, 2020–2024 [11,12,13,14,15].

Human Cases	Year				
	2020	2021	2022	2023	2024
Cases of illness	1876	2183	2738	2952	3041
Hospitalizations	780	923	1330	1497	1715
Deaths	167	196	286	335	301
Notification rates *	0.42	0.49	0.62	0.66	0.69
Infections acquired in the EU	1285	1482	1778	2031	2062
Infections acquired outside of the EU	5	4	12	8	14
Unknown travel/unknown country of infection	586	697	948	913	965
Total number of outbreaks	16	23	35	19	38

Legend: * = per 100,000 population.

**Table 2:** Comparison of *L. monocytogenes* regulatory frameworks.

Regulatory Framework	Risk Philosophy	*L. monocytogenes* Limits (RTE)	Risk Management and Criteria	References
US (USDA-FSIS)	Zero-tolerance Lm = hazard; product adulterated if detected	Absence (not detected); detection triggers rework or destruction	Alternative 1–3 verification intensity based on PLTs and AMAPs	[59]
US (FDA)	Zero-tolerance—focus on environmental monitoring as primary control	Not detected (< 1 cfu/25 g); goal is consistent destruction of viable cells	Zone System (Zones 1–4) Monitoring based on proximity to FCSs	[60]
Canada (Health Canada)	Risk-based approach—RTE foods classified potential to support Lm growth	Categorised limits— Category 1 (supports growth): absence in 25 g; Category 2 (limited/no growth): limit of 100 cfu/g	Enforcement—sale considered contravention if limits exceeded; focus on environmental sampling in post-process	[61]
European Union (EC)	Preventive approach—distinguishes Food Safety Criteria (market) and Process Hygiene Criteria (production)	Absence in 25 g (Infants/Medical) or 100 cfu/g during shelf life if growth restricted; otherwise, absence in 25 g	FBOs responsibility—must conduct shelf life studies (Annex II) to ensure 100 cfu/g limit is not exceeded	[22,23]
Australia and New Zealand (FSANZ)	Risk-based approach—occasional low-level contamination may be unavoidable if growth not supported	Not detected in 25 g (if growth can occur); 100 cfu/g (if growth will not occur)	By growth potential—two endpoint values depending on food’s ability to support growth	[62]
China (NHCPRC)	Mandatory limits—FBOs must minimise pathogen risks in prepackaged foods	Not detected in 25 g (meat, dairy, vegetables/fruits, frozen drinks, aquatic products)	Sampling plans—uses *n*, c, m, and M (typically *n* = 5, c = 0) to define safety limits	[63]
United Kingdom (UKHSA)	Public health focus—guidelines complement statutory laws for interpreting results	Not detected in 25 g (Infants/Medical); satisfactory if < 20 cfu/g; unsatisfactory if >100 cfu/g	Sampling plans— 2-class plans for safety; 3-class plans (satisfactory/borderline/unsatisfactory) for indicators	[64]
Japan (MHLW)	Public health focus—primary responsibility on FBOs; safety ensured through voluntary inspections	≤100 cfu/g for natural cheeses and non-heat-treated meat products; sale prohibited if contaminated	Approval system for comprehensive sanitation management; mandatory; Food Sanitation Supervisors for dairy and additives	[65,66]

Acronyms: USDA-FSIS = United States Department of Agriculture—Food and Safety Inspection Service; FDA = Food and Drug Administration; EC = European Commission; FSANZ = Food Standards Australia and New Zealand; NHCPRC = National Health Commission of the People’s Republic of China; UKHSA = UK Health Security Agency; MHLW = Ministry of Health, Labor and Welfare; PLTs = post-lethal treatments; AMAPs = antimicrobial agent or processes; RTE = ready-to-eat; FBOs = food business operators; FCSs = food contact surfaces.

**Table 3:** Main differences between the goals of challenge testing in the EU and US.

Agency	Primary Goal/Concept	Inhibition/Listeriostatic Goal	Lethality/Listericidal Goal
EURL Lm (EU)	Validate shelf life of RTE food and determine Lm behaviour. Foods are categorised based on their ability to support growth	Growth potential (δ) must be less than or equal to 0.5 log_10_ cfu/g throughout the shelf life to be deemed unable to support growth	Not the primary metric. Focus on survival or growth potential rather than specific log reduction
FDA (US)	Validate listeriostatic formulations or listericidal processes, demonstrating compliance with regulatory limits	Less than a 1-log increase in Lm numbers throughout the product shelf life (replicate studies)	The process should consistently reduce Lm to less than 0.04 cfu/g (< 1 cfu/25 g). A 5-log to 6-log reduction may be needed for inactivation validation
USDA-FSIS (US)	Provide scientific justification for Post-Lethality Treatments (PLTs) and Antimicrobial Agents/Processes (AMAPs) to comply with the *Listeria* Rule (9 CFR §430)	No more than 2-log growth of Lm over the shelf life to be considered effective (for AMAP documentation)	At least a 1-log reduction before leaving the establishment (for PLT). For reprocessing contaminated product, at least a 5-log reduction is expected

**Table 4:** Comparison between European and US challenge test protocols.

Feature	EURL Lm Technical Guidance Document (TGD)	FDA Approach [70]
Primary Focus and Study Types	Focuses on assessing RTE shelf life based on Lm growth and survival. Defines specific tests: Growth Potential (δ), Maximum Growth Rate (μmax), and Durability Study (verification of shelf life)	Describes challenge testing broadly as a tool to determine if a product supports growth (inhibition) or to validate lethal effects (inactivation of Lm by processes)
Strain Usage (δ test)	Requires a mixture of Lm strains (cocktail) to account for strain variability	Recommends using 3–5 strains of Lm, either individually or in combination, though a multiple-strain inoculum is usually preferred
Strain Usage (μmax test)	Typically uses a single strain per test, preferably one with known cardinal values	Allows screening to use a single, most resistant strain, but usually prefers a multiple-strain inoculum (3–5 strains)
Inoculum Level (Growth Studies)	Targets a contamination level of around 100 cfu/g (range 50 to 200 cfu/g) to minimise measurement uncertainty at low numbers	Typically uses an inoculum level between 100 and 1000 cfu/g of product to determine if a formulation supports growth
Inoculum Level (Lethality Studies)	Protocols focus on growth; lethality challenges are not a primary focus	Requires a high inoculum level (e.g., 10^6^–10^7^ cfu/g) for validating high-level lethality (like heat processing) to demonstrate the extent of reduction
Storage Conditions	Must reflect the foreseeable temperature range along the cold chain (e.g., multi-step time/temperature profiles) for growth potential (δ). μmax tests are conducted at one constant temperature (6 to 10 °C)	Often conducted at more than one temperature (e.g., 4.4–7 °C and 10–12 °C) or may incorporate temperature storage variations to simulate cold chain abuse
Duration and Sampling Frequency	The test period finishes at the end of the shelf life. Requires at least 4 sampling points (excluding t_0_)	Should extend over at least the desired shelf life, and preferably for the shelf life plus an additional margin (e.g., 1.25–1.5 times the length). Generally, requires a minimum of 5–7 sample intervals over the shelf life
Acceptance Criteria (Non-Growth)	A food is classified as unable to support growth if the growth potential (δ) is lower than or equal to 0.5 log_10_ cfu/g	An appropriate acceptance criterion for non-growth is a <1 log increase above the initial inoculum level throughout the shelf life
Laboratory Analytical Methods and Standards	Microbiological reference methods: EN ISO 11290-1 [71] (Detection) and EN ISO 11290-2 [72] (Enumeration). Recommends lowering the limit of enumeration to 10 cfu/g. Physical and chemical measurements: standard measurement of pH and a_w_; water phase salt content (WPS) to estimate a_w_ if necessary. Modelling with commercial software (e.g., DMFit from ComBase or Sym’Previus) for fitting primary models to estimate μmax	Microbiological reference methods: Refers to the FDA (BAM) and USDA FSIS methods. Discusses using non-selective media but lists various selective chromogenic plating media (e.g., ALOA^®^, CHROMagar *Listeria*) for enumeration. Injured Cell Recovery: resuscitation steps or overlay methods may be required for cells injured by lethal treatments or antimicrobials. Physical and chemical measurements involve tracking product parameters like pH, a_w_, preservatives, salt level, and gas concentrations (for MAP) using standard methods

**Table 5:** Differences between official laboratory methods for the detection and enumeration of *Listeria* spp. and *L*. *monocytogenes*.

Procedural Aspects	EN ISO 11290-1	EN ISO 11290-2	USDA FSIS MLG 8.15	FDA BAM Chapter 10
Primary Target	Detection of Lm in food and food processing environments	Enumeration of Lm in food and food processing environments	Isolation and identification of Lm and *Listeria* spp. in RTE products and environmental samples	Detection and enumeration of Lm in foods and environmental samples
Enrichment Medium	Half-Fraser broth	Only initial suspension is required. Diluents like BPW or Half-Fraser broth base can be used	Uses BioMèrieux^®^ LPT *Listeria* enrichment broth in a single-step enrichment process, replacing older two-step methods (UVM Broth and Morpholinepropanesulfonic acid-BLEB)	BLEB
Enrichment Procedure	Two steps: Primary enrichment in Half-Fraser (25 ± 1 h), followed by secondary enrichment in Fraser broth (reduced to 24 h duration). Refrigeration of broths or isolation plates is permissible before transfer or reading	No enrichment step required for enumeration/quantification methods	Single step: Incubation typically at 35±1 °C for 22–26 h for food or 18–24 h for environmental samples	Two steps: Basal BLEB (4 h at 30 °C), followed by addition of selective agents (acriflavin, cycloheximide, nalidixic acid), and further incubation at 30 °C for 24 to 48 h
Isolation Media	ALOA is the standard medium, often used alongside a second selective agar of choice (e.g., PALCAM, Oxford, RAPID’L.mono)	ALOA. Typical blue–green colonies with a white halo (*L. monocytogenes* or *L. ivanovii*). Plates containing < 100 characteristic colonies are preferred	HLCA replaced HBO agar and MOX agar	Esculin-based selective agars (e.g., Oxford, PALCAM, MOX, LPM) and chromogenic differential agars (*L. monocytogenes*-*L. ivanovii* differential agars, e.g., ALOA, R&F LMCPM, RAPID’L.mono)
Primary Screening/ Detection	Positive samples are most often detected after 24 h of Half-Fraser enrichment, but secondary enrichment is kept for efficiency, especially with highly stressed cells or competing microbial populations	Enumeration is achieved by surface plating decimal dilutions directly onto selective agar (ALOA)	Rapid screening of enriched samples for *Listeria* spp. using Neogen^®^ Molecular Detection Assay 2 (PCR technology). Positive enrichments are then plated onto HLCA	Qualitative detection methods generally involve enrichment followed by isolation/identification. Alternative rapid methodologies (e.g., AOAC OMA kits, PCR) may be used for screening. Enumeration is carried through MPN or direct plating
Confirmation/ Identification	Classical tests (Gram stain, Haemolysis, Sugar Utilisation). Catalase and CAMP test are optional, unless required by commercial miniaturised galleries. All mandatory tests must be performed if using commercial kits	Classical tests are used for confirmation, though the Catalase and CAMP test became optional in the revised standard. Commercial biochemical galleries may be used	Proteomic confirmation: Bruker^®^ MALDI Biotyper is the primary method, confirming isolates from Sheep Blood Agar (SBA) plates. PCR is used for screening presumptive *L. monocytogenes* colonies from HLCA	Standard: Haemolysis, CAMP test, Motility, Catalase, Gram stain, carbohydrate fermentation series. Alternate: Rapid biochemical kits (e.g., API^®^ *Listeria*, MICRO-ID™) or real-time PCR

Acronyms: BPW = buffered peptone water; BLEB = buffered *Listeria* enrichment broth; UVM = university of Vermont; ALOA: agar *Listeria* according to Ottaviani and Agosti; HLCA = Harlequin® *Listeria* chromogenic agar; HBO = horse blood overlay; MOX = modified Oxford; LMCPM = *Listeria monocytogenes* chromogenic plating medium; SBA = sheep blood agar; RTE = ready-to-eat; PCR = polymerase chain reaction.

**Table 6:** Summary and comparison between conventional and advanced detection methods.

Method Category	Specific Technique	Key Principle	Primary Advantages	Main Limitations	References
Conventional	Culture-Based Plating	Growth on selective and differential agar media followed by biochemical tests	Considered the gold standard; cost-effective; confirms the presence of viable cells	Very slow turnaround time (18–24 h to several days); labour-intensive; cannot detect VBNC (viable but non-culturable) cells	[82,83]
Conventional	Standard ELISA	Antibody–antigen interaction detected via enzymatic colour changes on a microtiter plate	High throughput; enables the detection of bacterial toxins; less expensive than molecular methods	Subject to false positives due to cross-reactivity; requires specific antibody purity; limited sensitivity	[82,83,84]
Conventional	Standard PCR	Amplification of specific DNA fragments through thermal cycling (denaturation, annealing, extension)	Highly sensitive and specific; provides reliable results for genetic identification	Requires expensive equipment and specialised personnel; cannot distinguish between live and dead cells without viability dyes	[81,82,83]
Advanced	Isothermal (LAMP)	DNA amplification at a constant temperature (60–65 °C) using four to six primers	No thermal cycler needed; results often obtained in under one hour; high amplification efficiency	Primer design is complex; high risk of false-positive results if ring primers are used incorrectly	[82,83,84]
Advanced	Biosensors (Optical/Electrochemical)	Bioreceptors (antibodies, aptamers, or enzymes) coupled with transducers to produce a measurable signal	Rapid, real-time results; high specificity; portable; allows for small sample volumes	High initial instrument and software costs	[81,82,83]
Advanced	CRISPR-Cas-Based	Utilising Cas enzymes (Cas12/13) to target and cleave specific nucleic acid sequences	Extraordinary specificity for single nucleotide polymorphisms; operates at physiological temperatures; rapid detection	Requires further optimisation for complex food matrices; quantitative analysis can be complex	[82,83]
Advanced (New)	Mass Spectrometry (MALDI-TOF MS)	Identifying pathogens by analysing characteristic ions and protein fingerprints of ionised cells	Highest detection rate; extremely fast operation; avoids total dependence on traditional microbial databases	Very high initial instrument cost; requires constant calibration of flow rate and spray voltage	[84]

## Data Availability

The original contributions presented in this study are included in the article. Further inquiries can be directed to the corresponding authors.
